# Dynamic Expression of HDAC3 in db/db Mouse RGCs and Its Relationship with Apoptosis and Autophagy

**DOI:** 10.1155/2020/6086780

**Published:** 2020-03-01

**Authors:** Yuhong Fu, Ying Wang, Xinyuan Gao, Huiyao Li, Yue Yuan

**Affiliations:** Department of Endocrinology, The First Affiliated Hospital of Harbin Medical University, Harbin 150001, China

## Abstract

**Background:**

Diabetic retinopathy (DR) is a severe complication of diabetes mellitus. DR is considered as a neurovascular disease. Retinal ganglion cell (RGC) loss plays an important role in the vision function disorder of diabetic patients. Histone deacetylase3 (HDAC3) is closely related to injury repair and nerve regeneration. The correlation between HDAC3 and retinal ganglion cells in diabetic retinopathy is still unclear yet.

**Methods:**

To investigate the chronological sequence of the abnormalities of retinal ganglion cells in diabetic retinopathy, we choose 15 male db/db mice (aged 8 weeks, 12 weeks, 16 weeks, 18 weeks, and 25 weeks; each group had 3 mice) as diabetic groups and 3 male db/m mice (aged 8 weeks) as the control group. In this study, we examined the morphological and immunohistochemical changes of HDAC3, Caspase3, and LC3B in a sequential manner by characterizing the process of retinal ganglion cell variation.

**Results:**

Blood glucose levels and body weights of db/db mice were significantly higher than that of the control group, *P* < 0.01. Compared with the control group, the number of retinal ganglion cells decreased with the duration of disease increasing. HDAC3 expression gradually increased in RGCs of db/db mice. Caspase3 expression gradually accelerated in RGCs of db/db mice. LC3B expression dynamically changed in RGCs of db/db mice. HDAC3 was positively correlated with Caspase3 expression (*r* = 0.7424), *P* < 0.01. Compared with the control group, the number of retinal ganglion cells decreased with the duration of disease increasing. HDAC3 expression gradually increased in RGCs of db/db mice. Caspase3 expression gradually accelerated in RGCs of db/db mice. LC3B expression dynamically changed in RGCs of db/db mice. HDAC3 was positively correlated with Caspase3 expression (*r* = 0.7424), *P* < 0.01. Compared with the control group, the number of retinal ganglion cells decreased with the duration of disease increasing. HDAC3 expression gradually increased in RGCs of db/db mice. Caspase3 expression gradually accelerated in RGCs of db/db mice. LC3B expression dynamically changed in RGCs of db/db mice. HDAC3 was positively correlated with Caspase3 expression (*Discussion*. We clarified the dynamic expression changes of HDAC3, Caspase3, and LC3B in retinal ganglion cells of db/db mice. Our results suggest the HDAC3 expression has a positive correlation with apoptosis and autophagy.

## 1. Introduction

Diabetic retinopathy(DR) is a common and severe complication of diabetes mellitus. In 2017, the American Diabetes Association (ADA) defined DR as a highly tissue-specific neurovascular complication. In the recent years, lots of clinical electrophysiological studies [1] have shown that before the clinical onset of DR in diabetic patients, the amplitude of the oscillating potential electroretinogram wave originated from the inner layer of neuroretina decreased and the latency period was prolonged, which was abnormally earlier than the abnormal blood-retinal barrier permeability. Retinal ganglion cells (RGCs) play a particular important role in the visual function of diabetic patients. Studies have been proved that RGC numbers and function changed in DR [2]. Moreover, it has been found that before the occurrence of retinal microvascular diseases in diabetic patients, there has been an obvious decline of the thickness of ganglion cell layer in neuroretina [3].

The pathogenesis of diabetic retinopathy is a complex, multiple factor process, including hyperglycemia, oxidative stress, advanced glycation end products (AGEs), protein kinase C, and inflammation. Nevertheless, the exact mechanism of DR is still not clear. Recently, evidences [4] suggested that apoptosis and autophagy in retinal ganglion cells have close associations with the pathogenesis of DR.

It has been widely approved that histone modification plays a central role in controlling gene expression and regulating disease. Histone acetylation is a major modification that affects gene transcription and required for many aspects of mammalian development and physiology, including injury repairment and nerve regeneration. Histone acetyl transferases (HATs) and histone deacetylases (HDACs) are two antagonistic enzyme families, which regulate the histone acetylation. HATs add acetyl groups to specific lysine residues, which result in relaxing of chromatin structure and facilitating gene activation, whereas HDACs are responsible for removing acetyl groups from hyperacetylated histones and suppress gene transcription. In addition to histones, numerous nonhistone proteins can be acetylated and deacetylated, and they also are involved in the regulation of a wide range of diseases [5]. Based on the molecular similarities with yeast homologues, HDACs are grouped into four classes [6]. Class I includes HDACs 1, 2, 3, and 8, primarily located at nuclear; Class II includes HDACs 4, 5, 6, 7, 9, and 10, which shuttle between nucleus and cytoplasm; Class IV is only comprised of HDAC 11. Class III is constituted of NAD^+^-dependent sirtuins, including SIRTs 1, 2, 3, 4, 5, 6, and 7 [7, 8].

Growing evidences indicated that HDAC3 is related to diabetes and the complications, including diabetic liver lesions, diabetic aortic atherosclerosis [9], diabetic osteoporosis [10], diabetic cardiomyopathy, diabetic cerebral ischemia reperfusion model [11], and diabetic nephropathy model [12]. Meanwhile, HDAC3 activity has been associated with the apoptosis of RGCs in models of optic nerve damage [13]. HDAC3 inhibition could reduce apoptosis and enhance autophagy in a diabetic cerebral I/R injury model [11]. However, the correlation between HDAC3 and retinal ganglion cells in diabetic retinopathy is still unclear yet.

Db/db mouse has been widely used as a T2DM model to investigate the pathogenesis of DR [14]. In this study, we examined the morphological and immunohistochemical changes of HDAC3, Caspase3, and LC3B in a sequential manner (8, 12, 16, 18, and 25 weeks) by characterizing the process of retinal ganglion cell variation.

## 2. Materials and Methods

### 2.1. Animals and Reagents

A lot of studies showed that the body weight and blood glucose levels in db/m mice did not change significantly with the increase of duration nor did the thickness of ganglion cell layer and the percentage of apoptosis and the percentage of autophagy show significant changes [15, 16]. Therefore, we selected db/m 8-week-old mice as the control group. To investigate the chronological sequence of the abnormalities of retinal ganglion cells in diabetic retinopathy, 15 male db/db mice (aged 8 weeks, 12 weeks, 16 weeks, 18 weeks, and 25 weeks; each group had 3 mice) as diabetic groups and 3 male db/m mice (aged 8 weeks) as the control group were purchased from Experiment Animal Center of Nanjing Medical University (Jiangsu, China). All mice were housed under controlled conditions of temperature at 20°C and humidity at 60%, with a 12-hour light/dark cycle and water and food ad libitum. Blood glucose concentrations were measured from the tail vein. Body weight were recorded. The study was approved by the Ethics Committee of First Affiliated Hospital of Harbin Medical University (approval number: 201798). HDAC3 antibody (YP0921) and LC3B antibody (YM3881) were purchased from ImmunoWay Biotechnology Company Inc. (Plano, Texas, USA). Caspase3 antibody (BA2142) was purchased from Boster Biological Technology Co. Ltd (Pleasanton, California, USA).

### 2.2. HE Staining

Retinal tissues were selected from random eye of each mouse in each group. The specimens were embedded in paraffin, afterwards cut into sections with 5-micron thickness, stained with hematoxylin and eosin (HE) by a series of standard techniques. Histological morphometric changes were observed under a light microscope. Image analysis of 3 sections from the same distance away from the optic nerve in each region was used to quantify total retinal thickness and RGC number per mm^2^.

### 2.3. Immunohistochemistry

Retinal tissues were selected from random eye of each mouse in each group. Paraffined sections were cut into sections with 5 microns, dewaxed and hydrated, and incubated with 3% hydrogen peroxide solution in dark boxes at room temperature for 25 minutes. Subsequently, the antigen was repaired by microwave, and sections were washed with PBS for 3 times. The sections were incubated in BSA for 20 minutes at room temperature and incubated with Caspase3 antibody (1 : 50), LC3B antibody (1 : 100), and HDAC3 antibody (1 : 20) overnight at 4°C, respectively. After that, the ocular sections were washed 3 times with PBS, each time for 5 minutes, and stained by DAB using secondary antibody kits. The staining time was controlled by evaluation under the microscope, then stopped by distilled water rinsing. Finally, tissues were restained by hematoxylin for 3 minutes and then washed, dehydrated, disposed with xylene, and coverslipped. The results were observed by light microscopy. Image analysis of 3 sections from the same distance away from the optic nerve in each region was used to quantify the percentage of immunohistochemical positive retinal ganglion cells in the total numbers of RGCs.

### 2.4. Statistical Analysis

All measurement data were expressed as mean ± SD, one-way analysis of variance (ANOVA) was used for comparison of all data, *q* test was used for comparison between groups, and linear correlation analysis was used for the correlation between indicators. *P* < 0.05 was considered statistically significant.

## 3. Results

### 3.1. Blood Glucose Level and Body Weight

As shown in Table 1, blood glucose levels of db/db mice were significantly higher than that of the db/m group (27.6 ± 3.2130 mmol/L vs. 5.6 ± 1.0817 mmol/L; *P* < 0.01); the body weights of the db/db mouse group were also significantly higher than that of the control group (40.4 ± 1.2288 g vs. 19.2 ± 1.8009 g; *P* < 0.01). These results indicated that the type 2 diabetic db/db mouse models were established successfully at 8 weeks.

### 3.2. Morphometric Observation of Retinal Tissues

**Figure 1:**
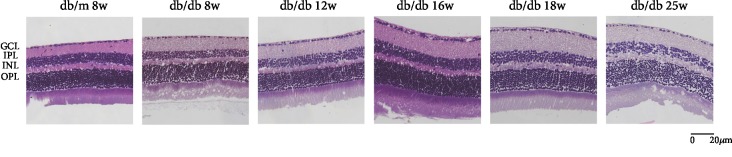
Photomicrographs of retinas from mice with hematoxylin-eosin (HE) staining of six groups: (a) the control group, db/m mouse at 8 weeks; (b) db/db mouse at 8 weeks; (c) db.db mouse at 12 weeks; (d) db/db mouse at 16 weeks; (e) db/db mouse at 18 weeks; and (f) db/db mouse at 25 weeks. The scale bar represents 20 *μ*m.

**Figure 2:**
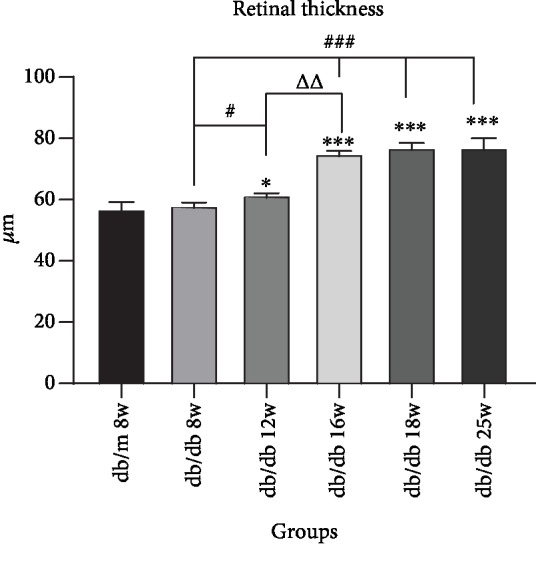
Augments of retina thickness in sequential diabetic mouse retinas. Annotation: compared with db/m mice at 8 weeks: ^∗^*P* < 0.05, ^∗∗^*P* < 0.01; compared with the last db/db mouse group: *^Δ^P* < 0.05, *^ΔΔ^P* < 0.01.

**Figure 3:**
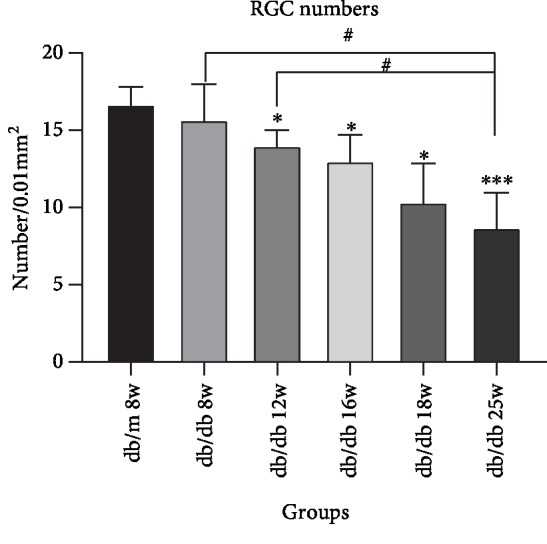
Decrease of RGC numbers in sequential diabetic mouse retinas. Annotation: compared with db/m mice at 8 weeks: ^∗^*P* < 0.05, ^∗∗^*P* < 0.01; compared with the other db/db mouse group: ^#^*P* < 0.05.

Morphological changes of HE-stained retinal ganglion cells and layers of retina had been observed by an optical microscope. In Figure 1, HE staining showed that under a light microscope the retinal surface of the control group was smooth, the morphology of each layer was normal, the structure was clear and complete, and the cells were closely arranged. Compared with the control group, the retinal structure in the diabetic group was gradually irregular, the thickness of retina increased, each layer of the retina became significantly loose and disordered, and the number of retinal ganglion cells decreased with the duration of disease increasing. We analyzed the thickness of central retina in Figure 2, which showed that the thickness gradually increased. Compared with the db/m group, there was no significant difference between db/db 8 weeks, while db/db 12 weeks had a statistical significance with db/m mice, which is *P* < 0.05. And db/db 16 weeks, 18 weeks, and 25 weeks had a significant statistical significance compared with that in the control group, which is *P* < 0.01. In Figure 3, the number of RGCs decreased as the duration of disease progressed. Compared with the control group, db/db 12 weeks, 16 weeks, and 18 weeks had a statistical significance, which is *P* < 0.05. And the db/db 25-week group had a significant statistical significance with the db/m 8-week group, which is *P* < 0.01. Meanwhile, in diabetic groups, the number of RGC in the db/db 25-week group had a statistical significance between db/db 8 weeks and db/db 12 weeks, which is *P* < 0.05. The results showed the morphological changes with the duration of diabetes that the retina thickness increased and the RGCs decreased.

### 3.3. HDAC3 Expression Gradually Increased in RGCs of db/db Mice

**Figure 4:**
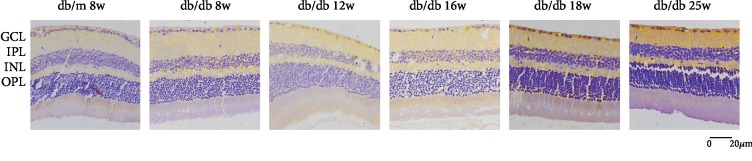
Immunostaining of HDAC3 in db/db mice and db/m mice of six groups: (a) the control group, db/m mouse at 8 weeks; (b) db/db mouse at 8 weeks; (c) db.db mouse at 12 weeks; (d) db/db mouse at 16 weeks; (e) db/db mouse at 18 weeks; and (f) db/db mouse at 25 weeks. The scale bar represents 20 *μ*m.

**Figure 5:**
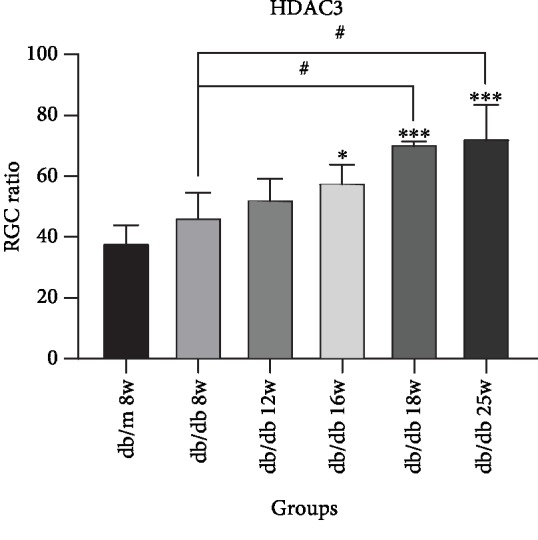
Augments of HDAC3 expression in sequential diabetic mouse retinas. Annotation: compared with db/m mice at 8 weeks: ^∗^*P* < 0.05, ^∗∗^*P* < 0.01; compared with the other db/db mouse group: ^#^*P* < 0.01.

In Figure 4, we could find that HDAC3 expresses in RGC nucleus. Compared with the control group, the expression of HDAC3 increased in RGCs at 8 weeks and 12 weeks in the early DR stage, but there was no statistical significance in Figure 5. In the middle and late DR stage, HDAC3 expression increased at 16 weeks, 18 weeks, and 25 weeks, with statistical significance. Comparing with HDAC3 expression in db/db mice at 8 weeks, the expression of 18 weeks and 25 weeks both had statistical augments, which are *P* < 0.01. The expression of HDAC3 increased between 16 weeks and 18 weeks, 12 weeks and 18 weeks, and 12 weeks and 25 weeks, showing a statistical significance, which is *P* < 0.05, while the expression of HDAC3 increased between 8 weeks and 12 weeks, 12 weeks and 16 weeks, and 18 weeks and 25 weeks, showing no statistical significance. These results suggested that HDAC3 expression increased with the duration of diabetes augmenting in RGCs of db/db mice.

### 3.4. Caspase3 Expression Gradually Accelerated in RGCs of db/db Mice

**Figure 6:**
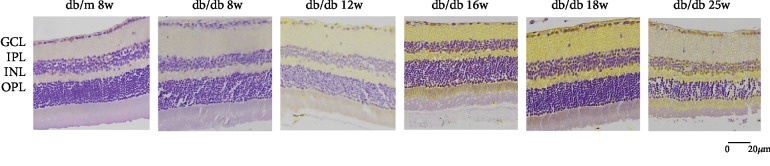
Immunostaining of Caspase3 in db/db mice and db/m mice of six groups: (a) the control group, db/m mouse at 8 weeks; (b) db/db mouse at 8 weeks; (c) db.db mouse at 12 weeks; (d) db/db mouse at 16 weeks; (e) db/db mouse at 18 weeks; and (f) db/db mouse at 25 weeks. The scale bar represents 20 *μ*m.

**Figure 7:**
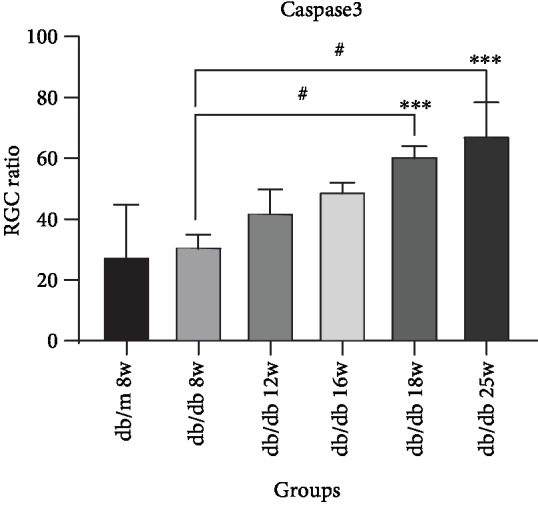
Augments of Capase3 expression in sequential diabetic mouse retinas. Annotation: compared with db/m mice at 8 weeks: ^∗^*P* < 0.05, ^∗∗^*P* < 0.01; compared with the other db/db mouse group: ^#^*P* < 0.01.

In Figure 6, we could find that Caspase3 expresses in RGC cytoplasm. Compared with the control group, the expression of Caspase3 increased in RGC at 8 weeks, 12 weeks, and 16 weeks in early and middle DR, but there was no statistical significance (Figure 7). In the middle and later DR stage, the expression of Caspase3 increased at 18 weeks and 25 weeks, which was statistically significant, *P* < 0.01. Comparing with Capase3 expression in db/db mice at 8 weeks, the expression of 18 weeks and 25 weeks both had statistical augments, which are *P* < 0.01. The expression increased rapidly from 8 weeks to 25 weeks. The expression of Caspase3 increased between 12 weeks and 25 weeks showing a statistical significance, which is *P* < 0.05. Compared between two sequential groups, 8 weeks and 12 weeks, 12 weeks and 16 weeks, 16 weeks and 18 weeks, and 18 weeks and 25 weeks, the Caspase3 expression was higher, but there was no statistical significance. These results suggested that Caspase3 expression increased with the duration of diabetes augmenting in RGCs of db/db mice.

### 3.5. LC3B Expression Dynamically Changed in RGCs of db/db Mice

**Figure 8:**
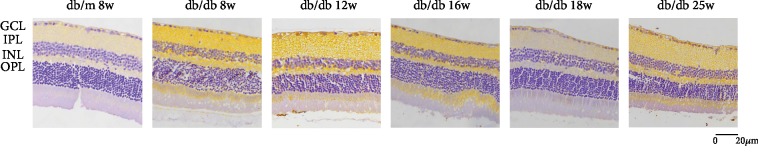
Immunostaining of LC3B in db/db mice and db/m mice of six groups: (a) the control group, db/m mouse at 8 weeks; (b) db/db mouse at 8 weeks; (c) db.db mouse at 12 weeks; (d) db/db mouse at 16 weeks; (e) db/db mouse at 18 weeks; and (f) db/db mouse at 25 weeks. The scale bar represents 20 *μ*m.

**Figure 9:**
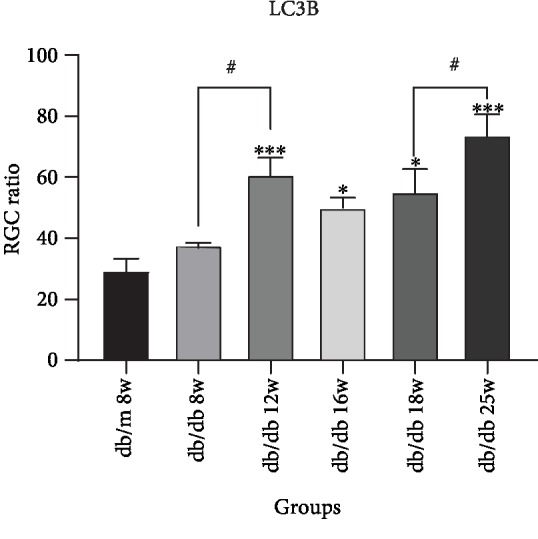
Augments of LC3B expression in sequential diabetic mouse retinas. Annotation: compared with db/m mice at 8 weeks: ^∗^*P* < 0.05, ^∗∗^*P* < 0.01; compared with the other db/db mouse group: ^#^*P* < 0.01.

In Figure 8, we could find that LC3B expresses in RGC cytoplasm. Compared with the control group, LC3B expression increased in RGC in the early stage of DR at 8 weeks, with no statistical significance. In the middle and late stage of DR, LC3B expression increased at 12 weeks, 16 weeks, 18 weeks, and 25 weeks, with statistical significance (Figure 9). Compared between 12 weeks and 8 weeks, 25 weeks and 18 weeks, LC3B expression increased in RGC with statistical significance, which is *P* < 0.01. In addition, compared with 16 weeks and 18 weeks, LC3B expression increased with no statistical significance. Moreover, compared with 16 w and 12 w, LC3B expression decreased. The results indicated that LC3B expression dynamically changed in RGCs of db/db mice.

### 3.6. The Correlation of HDAC3, Caspase3, and LC3B in RGCs of db/db Mice

**Figure 10:**
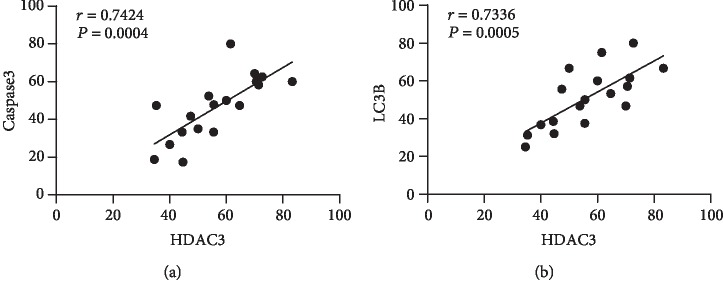
(a) Correlation of HDAC3 and Capase3, *r* = 0.7424, *P* < 0.01; (b) correlation of HDAC3 and LC3B, *r* = 0.7336, *P* < 0.01.

Linear analysis (Figure 10) showed that HDAC3 was positively correlated with Caspase3 expression (*r* = 0.7424), *P* < 0.01, which was statistically significant. HDAC3 was positively correlated with LC3B expression (*r* = 0.7336), *P* < 0.01, which was statistically significant. The results indicated that HDAC3 could be related to the apoptosis and autophagy of retinal ganglion cells in diabetic retinopathy.

## 4. Conclusions

C57BL/KsJ-db/db mouse is a sort of genetically defective mouse which has a mutation in the leptin gene receptor, widely used as a spontaneous diabetic rodent model [16]. In the past reports, db/db mice developed hyperinsulinemia at 10-14 days, followed by hyperphagia and obesity at about 4 weeks, and hyperglycemia at 4-8 weeks due to beta cell disorder, with the blood glucose peak at 3-4 months. db/db mice have similar symptoms to human type 2 diabetes, including polydipsia, polyphagia, polyuria, obesity, hyperglycemia, hyperinsulinemia, and abnormal lipid metabolism. Compared with the STZ-induced diabetes model, db mice are more stable and more similar to the human type 2 diabetes model; besides, streptozotocin (STZ) has neurotoxicity [14]. Moreover, as occurred in human retinas, retinal ganglion cell layer has been the layer with the highest rate of apoptosis in db/db mice [17]. Thus, db/db mouse seems to be an appropriate model to investigate the pathogenesis of diabetic retinopathy. Our results showed that at 8 weeks, db/db mice showed significant increases in the blood glucose level and body weight compared with the control group, consistent with previous results. In this study, we examined the morphological and immunohistochemical changes in a sequential manner (8, 12, 16, 18, and 25 week) by characterizing the process of retinal ganglion cell variation. Caspase3 and LC3B, which were widely used as the biomarkers of apoptosis and autophagy, respectively, were investigated via immunohistochemistry analysis in this research. We observed that HDAC3, Caspase3, and LC3B expressions augmented along with the diabetes duration in RGCs of db/db mice.

HDACs are closely related to injury repair and nerve regeneration. Previous studies had proved that RGC apoptosis was the main form in the neuroretina injury of diabetic rats. The pathways of apoptosis mainly consist of endogenous mitochondrial pathway, endogenous endoplasmic reticulum stress-induced pathway, and exogenous death receptor pathway. Caspase3 is the most critical apoptotic protease in the downstream of Caspase cascade waterfall, which could initiate the internal mitochondrial apoptotic cascade reaction, thus leading to cell apoptosis [18]. It also regulates the activation of endoplasmic reticulum apoptosis pathway [19]. The conditional knockout of HDAC3 in RGCs resulted in obvious amelioration of optic nerve crush-induced nuclear atrophy, including Histone4 deacetylation, heterochromatin formation, and the loss of nuclear structure; RGC apoptosis was also apparently reduced [13]. Selective HDAC3 inhibitor RGFP966 has a high affinity for HDAC3 and a medium affinity for HDAC1 and HDAC2. A recent study mentioned that BBB (blood-brain barrier) permeability increased and early BBB destruction occurred at the age of 16 weeks in a type 2 diabetes db/db mouse model. HDAC3 expression was increased in the hippocampus and cortex of diabetic mice, and the blood-brain barrier permeability of db/db mice was decreased after the inhibition of HDAC3 activity by RGFP966, suggesting that HDAC3 may play a key role in T2DM-related neuronal diseases [20]. In 2018, some researchers found that HDAC3 regulates nuclear atrophy and apoptosis of retinal ganglion cells (RGCs) following optic nerve crush (ONC). Histone deacetylation and RGC survival rates were improved after application of low levels of RGFP966, which is possibly due to the different binding rates and absorption of RGFP966 to systemic targets prior to its passage through the blood-retinal barrier. HDAC3 may be a potential target for treatment of the injury of RGCs [21]. But neither of them studied about the related change of HDAC3 expression of RGCs in DR stage and the relationship between apoptosis and autophagy. Our result showed that HDAC3 had a positive relationship with Caspase3 in RGCs of DR stage. Increased expression of Caspase3 indicates the occurrence of apoptosis in retinal ganglion cells. To our best knowledge, it is the first report on the positive correlation between HDAC3 and apoptosis of the RGCs in diabetic retinopathy.

Autophagy is also closely related to DR. Autophagy is a catabolic pathway that facilitates the cellular component degradation and recycling in lysosome, meanwhile as an adaptive mechanism to protect cells against stress-induced injury. Reactive oxygen species (ROS) produced by mitochondria was a kind of metabolic abnormalities, which was caused by hyperglycemia [22]. ROS can induce oxidative stress augments. Oxidative stress is one of the crucial pathological factors in the pathogenesis of DR and is involved in the damage of RGCs in the early stage of DR [23]. Studies have shown that the mitochondrial density in RGCs is higher; thus, RGCs are more susceptible to oxidative stress in DR and then induce autophagy occurrence of RGCs [24]. Previous studies have confirmed that autophagy plays an important role in DR [25], which had a dual role in regulating cell survival. In human retinal capillary pericytes (HRCPs), mild stress induced protective autophagy and ER stress but not apoptosis, while under severe stress, autophagy promotes cell death. RGC neuropathy is associated with autophagy in the early period of the DR. Piano et al. [26] have observed increased autophagy levels in the retinal ganglion cell layer of STZ-induced diabetic mice.

HDAC3 plays a crucial role in autophagy response of neuron injury. It has been shown that HDAC3 expression increased in brain neurons of traumatic brain injury (TBI) [27]. Mitochondria release more ROS in damaged brain neurons, activates oxidative stress and inflammation, and then induces selective autophagy to reduce oxidative damage to maintain the normal function of mitochondria and balance the intracellular microenvironment. Retinal neurons are brain-derived tissues. Several pathways that trigger neurodegenerative diseases in the brain, such as impaired insulin signaling, inflammation, accumulations of advanced glycation end products (AGEs), and oxidative stress, could also trigger retinal neuropathy of type 2 diabetes mellitus patients [28]. In a diabetic cerebral ischemia/reperfusion injury mouse model [11], HDAC3 inhibition upregulated autophagy level and attenuated cell apoptosis. Our results indicated that autophagy was activated in diabetic retinal ganglion cells. Besides, the middle stage level of autophagy decreased than the earlier stage, which may be due to the imbalance of protection and destruction autophagy function. Our study showed a positive correlation between HDAC3 and apoptosis of the retinal ganglion, though the exact mechanism remains to be further studied.

So far, HDAC3 has attracted a lot of attention of scholars. It has been demonstrated playing a vital role in injury repair and nerve regeneration. Focus on its multifunctional characteristics could broaden the scope for the treatment of clinical diseases. However, till now, there are few researches about HDAC3 and retinal ganglion cell injury in DR. We clarified the dynamic expression changes of HDAC3, Caspase3, and LC3B in retinal ganglion cells of db/db mice. Our results suggest the HDAC3 expression has positive correlation with apoptosis and autophagy. The findings reveal the prospects of HDAC3 in the treatment of DR and lay a theoretical foundation for its clinical application. Our experiment has several limitations. We should set a HDAC3-inhibited control group in order to observe that the inhibition of HDAC3 expression would inhibit RGC apoptosis and autophagy in DR or activate the progression. In addition, the underlying molecular pathway of HDAC3 with apoptosis and autophagy is still unclear. Hence, we expect to continue relevant in vivo and in vitro intervention researches in the future work.

## Figures and Tables

**Table 1:** Blood glucose level and body weight in aged 8 weeks db/db mice and db/m mice (*x* ± *s*, *n* = 3).

Groups	Blood glucose (mmol/L)	Body weight (g)
db/m	5.6 ± 1.0817	19.2 ± 1.8009
db/db	27.6±3.2130^∗∗∗^	40.4±1.2288^∗∗∗^

Annotation: compared with aged 8 weeks db/db mice, ^∗^*P* < 0.05, ^∗∗^*P* < 0.01.

## Data Availability

The data used to support the findings of this study are included within the article.
